# Impact of COVID-19 on patients treated with autologous hematopoietic stem cell transplantation: A retrospective cohort study

**DOI:** 10.48101/ujms.v127.8611

**Published:** 2022-08-25

**Authors:** Thomas Silfverberg, Björn Wahlin, Kristina Carlson, Honar Cherif

**Affiliations:** a Department of Medical Sciences, Uppsala University, Uppsala, Sweden; b Center for Clinical Research Dalarna, Uppsala University, Uppsala, Sweden; c Division of Hematology, Department of Medicine Huddinge, Karolinska Institutet, Stockholm, Sweden

**Keywords:** Hematopoietic stem cell transplantation, COVID-19, SARS-CoV-2 virus, multiple myeloma, lymphoma, immunosuppression therapy, transplantation autologous

## Abstract

**Objective:**

To describe how coronavirus disease 2019 (COVID-19) affects patients with hematological malignancies treated with autologous hematopoietic stem cell transplantation (ASCT).

**Methods:**

This retrospective observational cohort study includes all patients with hematological malignancies treated with ASCT in Sweden from 1 January 2020 to 31 December 2020. Patients who subsequently tested positive for severe acute respiratory syndrome coronavirus 2 (SARS-CoV-2) until 31 March 2021 were analyzed for morbidity, mortality, need for supportive care, and risk factors related to COVID-19.

**Results:**

This study identified 442 patients who underwent ASCT in Sweden in 2020, among whom 20 (4.5%) subsequently tested positive for COVID-19. The overall mortality was 15%, and the COVID-19-related mortality was 10% among the patients who contracted COVID-19. Six (35%) patients were hospitalized, of which four (24%) needed supplementary oxygen and two (12%) needed intensive care. The absolute risk of COVID-19-related mortality was 0.45%.

**Conclusions:**

ASCT patients have a higher risk of severe outcome of COVID-19 compared to the normal population. However, the risks of death, inpatient care, oxygen therapy, and intensive care seem lower in this study compared to previous studies, possibly due to fewer mildly ill patients in other studies. The risk of contracting SARS-CoV-2 appears to be comparable to that in the general population. This study suggests that the COVID-19 pandemic is not a strong argument for refraining from ASCT in the case of hematological malignancy.

## Introduction

Severe acute respiratory syndrome coronavirus 2 (SARS-CoV-2) is a novel coronavirus that emerged in the city of Wuhan, China, in the late 2019. The virus was isolated and described in January 2020 (1). During the following months, it caused a pandemic of coronavirus disease 2019 (COVID-19) affecting millions of people all over the world.

High-dose chemotherapy followed by autologous hematopoietic stem cell transplantation (ASCT) is a well-established treatment option for a wide range of hematological malignancies and some specific autoimmune disorders. Early studies of COVID-19 reported high mortality rates in patients who had a malignancy or who were immunocompromised (2, 3). The immunosuppression related to ASCT together with the risk of contracting severe SARS-CoV-2 led to modifications in the therapy recommendations and, in many cases, to postponed treatment (4, 5).

The burden of COVID-19 has been heterogeneous both nationally and internationally. Guidelines and policies to reduce the spread of infection have varied substantially. Sweden has been an outlier, as the country chose not to impose general lock-downs or curfews in the society, but rather relied on voluntary compliance, including recommendations that vulnerable people should self-isolate. Polymerase chain reaction (PCR) testing was limited due to inadequate availability until early June 2020, and self-isolation was proposed for people with mild symptoms.

In Sweden, several transplantation centers performed fewer ASCTs during the spring of 2020 at the peak of spread of the disease. ASCT for multiple myeloma was most affected, as alternative treatments could be given, postponing the ASCT to a later time.

There have been three waves of COVID-19 in Sweden, culminating in April 2020, December 2020, and April 2021 during the course of this study. The Wuhan strain of SARS-CoV-2 virus dominated the first two waves. In December 2020, the alpha-variant started spreading in Sweden and totally dominated the cases during the third wave. Since late 2020, there have also been low percentages of the beta-variant. By 4 April 2021, there were 831,561 cases and 13,531 deaths from COVID-19 (defined as death within 30 days of a SARS-CoV-2 positive PCR test or COVID-19 stated as cause of death) confirmed in Sweden (6). The median age of COVID-19-related deaths was 85 years, and 91% of the fatalities were over 70 years old (7). The total population in Sweden was 10,379,295 on 31 December 2020 (8), corresponding to an incidence rate of 8.0% and a COVID-19-related mortality rate of 1.63% (6).

We conducted a retrospective review of patients treated with ASCT due to hematological malignancy during 2020 to highlight the impact of the COVID-19 pandemic on this vulnerable group of patients.

## Methods

### Study design, setting, and data sources

This is a retrospective observational cohort study of all patients treated with ASCT for hematological malignancy in Sweden from 1 January 2020 to 31 December 2020. The local European Society for Blood and Marrow Transplantation (EBMT) registers at all seven university hospitals were used to identify patients together with basic data regarding age, gender, hematological diagnosis, and transplantation center. These hospitals are the only centers that perform ASCT. Patients who tested positive for SARS-CoV-2 from the start of conditioning until the end of the study on 31 March 2021 were included, allowing for at least 3 months of follow-up after ASCT. The relatively short time of follow-up after ASCT was chosen to evaluate the impact of the immunosuppression caused by the procedure in relation to COVID-19. After testing positive for SARS-CoV-2, patients were followed for a minimum of 3 months.

The Public Health Agency of Sweden (PHAS) has the national responsibility to surveil and control communicable diseases. The PHAS uses several different surveillance systems to monitor the spread of COVID-19. Since COVID-19 is subjected to mandatory reporting under the Communicable Diseases Act, physicians and laboratories continuously report data to the database SmiNet held by the PHAS. The coverage of this database is estimated to be very high, close to 100%. This study linked the patients identified through the local EBMT registers with SARS-CoV-2 positive patients in SmiNet.

Clinical data were obtained from systematic analysis of medical records. Mortality data were gathered at time of identification and/or analysis of the medical records.

#### Inclusion criteria

- Diagnosis of hematological cancer (C81–C96 according to the International Classification of Diseases 10th revision [ICD-10]).
- ASCT performed from 1 January 2020 to 31 December 2020 at a Swedish center.
- Positive real-time (RT)-PCR test for SARS-CoV-2 performed in Sweden from the start of conditioning until the end of the study period on 31 March 2021.

#### Exclusion criteria

- Age below 18 years at the time of transplantation.

### Study endpoints

The primary endpoints were overall survival at 30 and 90 days following a positive test for SARS-CoV-2 and COVID-19-related mortality.

Secondary endpoints included level of supportive care needed, occurrence of COVID-19-related complications, and risk factors for severe outcome (details in the Supplementary Material).

### Definition of data points

The World Health Organization’s definition for COVID-19-related mortality was used in this study. A death due to COVID-19 is defined as a death resulting from a confirmed COVID-19 case, unless there is a clear alternative cause of death that cannot be related to COVID-19 disease (e.g. trauma). There should be no period of complete recovery from COVID-19 between illness and death. A death due to COVID-19 may not be attributed to another disease (e.g. cancer) and should be counted independently of preexisting conditions that are suspected of triggering a severe course of COVID-19 (9).

Need for supportive care due to COVID-19 was defined as, at any time following infection with SARS-CoV-2 and during the course of active infection or its related complications, requiring admission to hospital or intensive care unit (ICU), as well as at any time requiring oxygen, high-flow oxygen therapy, non-invasive ventilation, invasive mechanical ventilation, or extracorporeal membrane oxygenation (ECMO). Occurrence of complications was defined as any diagnosis of specified complications in medical records, or findings through medical work-up, investigations or clinical signs clearly suggestive of a complication without an obvious other explanation. The level of supportive care was described using a modification of the WHO Clinical Progression Scale of viral infection (10).

### Statistical analysis

Descriptive statistics are used and results are expressed as median (range). Proportions are expressed with a 95% confidence interval (CI, Wilson Score) and incidences with 95% CI (Miettinen’s [1974d] modification of the Mid-P exact test) in OpenEpi, version 3.

## Results

In this study, 442 unique adult patients who underwent ASCT due to hematological malignancy were identified at the seven Swedish transplantation centers from 1 January 2020 to 31 December 2020. For characteristics of this cohort, see Table 1. A total of 20 patients (4.5% [CI: 2.95–6.89%]) with a median age of 60 (40.1–70.2) years subsequently tested positive for SARS-CoV-2 before the end of 31 March 2021. All patients were diagnosed using RT-PCR tests. Myeloma (13 patients) and B-cell lymphomas (four patients) were the most common underlying diseases, but there were also single cases of plasma cell leukemia, T-cell lymphoma, and Hodgkin’s lymphoma. The median time from ASCT to COVID-19 was 5.6 (0.6–11.6) months. Four patients (20%) had COVID-19 within the first 100 days following ASCT. None of the patients had been vaccinated for SARS-CoV-2 at time of infection. The cases were evenly distributed over the country regions, and no diagnosis was associated with a higher risk for infection. Symptoms of disease included fever, cough, and in one case headache. Median time from the start of symptoms until diagnosis of COVID-19 was 2 (0–7) days. Three patients (18%) were asymptomatic. Clinical data are missing for three patients from one center, except for data on age, mortality, and diagnosis, due to difficulties in clearing permission to obtain data. Characteristics of the ASCT-patients are summarized in Table 2.

**Table 1:** Characteristics of ASCT patients.

Variable	All ASCT	COVID-19–	COVID-19+
Total patients, *n* (%)	442	422 (95.5)	20 (0.45)
Sex			
Male	278 (62.9)	267 (63.3)	11 (55)
Female	164 (37.1)	155 (36.7)	9 (45)
Age (range 18.3–74.2 years)			
18–19	1 (0.2)	1 (0.2)	0
20–29	4 (0.9)	4 (0.9)	0
30–39	12 (2.7)	12 (2.8)	0
40–49	38 (8.6)	33 (7.8)	5 (25)
50–59	131 (29.6)	127 (30.1)	4 (20)
60–69	219 (49.5)	209 (51.9)	10 (50)
70-	37 (8.4)	36 (8.5)	1 (5)
Median, years	61.4	61.6	60.2
Hematological disease			
Myeloma	301 (68.1)	288 (68.2)	13 (65)
Other plasma cell disease	17 (3.8)	16 (3.8)	1 (5)
B-cell lymphoma	94 (21.3)	90 (21.3)	4 (20)
Hodgkin lymphoma	13 (2.9)	12 (2.8)	1 (5)
T-cell lymphoma	17 (3.8)	16 (3.8)	1 (5)
Transplantation center			
Karolinska	94 (21.2)	89 (21.1)	5 (25)
Linköping	45 (10.2)	41 (9.7)	4 (20)
Sahlgrenska	91 (20.6)	88 (20.9)	3 (15)
Skåne	74 (16.7)	71 (16.8)	3 (15)
Uppsala	66 (14.9)	62 (14.7)	4 (20)
Umeå	37 (8.4)	36 (8.5)	1 (5)
Örebro	35 (7.9)	35 (8.3)	0

ASCT: autologous hematopoietic stem cell transplantation.

The table shows the characteristics of all patients treated with hematopoietic autologous stem cell transplantation for hematological malignancy in Sweden in 2020 and how many of those later tested positive for SARS-CoV-2.

**Table 2:** Characteristics of COVID-19 positive ASCT patients.

Variable	Ambulatory (η:11)	Hospitalized (η: 3)^†^	Dead (η: 3)
Age, median (range)	60.1 (45.9–68.1)	57,0 (40.1–60.3)	65.3 (63.9–70.2)
Hematological disease, *n* (%)			
Myeloma	7 (64)	2 (67)	1 (33)
Plasma cell leukemia	1 (9)	-	-
B-cell lymphoma	2 (18)	1 (33)	1 (33)
Hodgkin lymphoma	1 (9)	-	-
Anaplastic T-cell lymphoma	-	-	1 (33)
Disease status at conditioning			
CR – complete remission	5 (45)	2 (67)	-
PR – partial response	6 (55)	1 (33)	2 (67)
SD – stable disease	-	-	1 (33)
PD – progressive disease	-	-	-
Comorbidities^‡^			
Diabetes	1 (9)	-	1 (33)
Hypertension	2 (18)	-	2 (67)
Chronic lung disease	-	-	1 (33)
Chronic kidney disease	-	1 (33)	-
Organ transplantation (kidney)	-	1 (33)	-
Obesity	1 (9)	2 (67)	-
No of prior lines of treatment			
1	9 (82)	1 (33)	2 (67)
2	-	2 (67)	-
5	2 (18)	-	1 (33)
Prior treatment (last 6 months)			
Tandem ASCT	2 (18)	1 (33)	-
Chemotherapy	3 (27)	2 (67)	2 (67)
Proteasome inhibitors	8 (73)	1 (33)	-
ImiDs	6 (55)	1 (33)	-
CD20 monoclonal antibodies	2 (18)	1 (33)	1 (33)
SLAMF7 monoclonal antibodies	-	-	1 (33)
Steroids	10 (91)	2 (67)	3 (100)
Radiotherapy	1 (9)	-	1 (33)
Immunotherapy	3 (27)	-	1 (33)
Brentuximab-vedotin	-	-	1 (33)
Conditioning			
Melphalan	8 (73)	2 (67)	1 (33)
BEAM	3 (27)	1 (33)	1 (33)
BEAC	-	-	1 (33)
Time of positive PCR after ASCT, median (range), months	5.6 (1.9–11.6)	8.6 (3.7–11.3)	8.7 (0.6–9.5)
Treatment given^§^			
Dexametason	-	-	2 (67)
Convalescent plasma	-	-	1 (33)
Remdesivir	-	1 (33)	-

IMIDS: immunomodulatory imide drugs; BEAM: carmustine, etoposide, cytarabine, and melphalan; BEAC: carmustine, etoposide, cytarabine, and cyclophosphamide; ASCT: autologous hematopoietic stem cell transplantation.

The table presents the characteristics of all patients treated with ASCT for hematological malignancy in Sweden in 2020 and tested positive for SARS-CoV-2. Clinical data, except for diagnosis and mortality, are missing for three patients. Percentages are shown for each column.

† Hospitalized patients who eventually died are excluded in this column.

‡ None of the patients in the cohort had a history of cardiovascular disease, stroke, chronic liver disease, neuromuscular disease, other active cancer, allogenic stem cell transplantation, or smoking.

§ No patients were given IL-6 pathway inhibitors, hydroxychloroquine, or baricinitinib.

Three patients died within the study period. All deaths occurred within 30 days of infection with SARS-CoV-2, corresponding to an overall mortality rate of 15.0% (CI: 5.24–36.0%). Two patients died from direct causes of COVID-19, and one patient died due to mantle cell lymphoma 5 days after testing positive. The first patient had stage four anaplastic lymphoma kinase negative anaplastic T-cell lymphoma, was infected when hospitalized at day 17 following the ASCT with BEAC (carmustine, etoposide, cytarabine, and cyclophosphamide), and was the only patient who was infected within the first month of transplantation. The patient was 70 years old and developed cytokine release syndrome (CRS), secondary bacterial infection, cytomegalovirus reactivation, and acute cardiac injury. The patient received two doses of convalescent plasma for SARS-CoV-2, off label, 26 days after the start of symptoms when in a deteriorating life-threatening condition with CRS and continued viral replication requiring intensive care and invasive respiratory assistance. The second patient had myeloma and had had five prior lines of treatment. The patient was infected at 64 years of age, more than 9 months after ASCT with melphalan, and developed acute respiratory distress syndrome (ARDS), CRS, and secondary infection. The third patient contracted SARS-CoV-2 almost 9 months after ASCT with BEAM (carmustine, etoposide, cytarabine, and melphalan) when already hospitalized and in an advanced palliative state due to relapse in mantle cell lymphoma. The patient had earlier received relapse treatment with bendamustine and rituximab. This patient did not develop severe symptoms of COVID-19, and the cause of death was determined as relapse in mantle cell lymphoma. COVID-19 was assessed not to have altered the outcome. The COVID-19-related mortality in this study was 10% (CI: 2.79–30.1%), and the absolute risk of COVID-19-related mortality following ASCT was 0.45% (CI: 0.12–1.63%).

The majority of infected patients (65% (CI: 41.3–82.7%) did not need hospitalization. Six patients (35% [CI: 17.3–58.7%]) were admitted to hospital; among these, four (24% [CI: 9.56–47.3%]) needed supplementary oxygen and two (12% [CI: 3.29–34.3%]) were admitted to the ICU, and they both died from COVID-19. No patient needed ECMO. The median duration of hospital stay was 8 (1–28) days. Clinical complications of COVID-19 were absent for all but the two patients who were admitted to the ICU, except for the one patient who developed a secondary bacterial infection. Four patients in this study received glucocorticoids as treatment for COVID-19, one received remdesivir, and one received convalescent plasma.

Comorbidities associated with severe outcome of COVID-19 were rare. Additional laboratory tests (see Appendix) were not performed on a general basis for non-hospitalized patients, and the number of tests taken and the abnormalities corresponded roughly to the outcome of COVID-19.

## Discussion

In this well-defined cohort of patients with hematological malignancies treated with ASCT, we describe an overall mortality of 15% and a COVID-19-related mortality of 10%, which seems lower when compared to previous studies (11–13). All deaths in the study were among patients older than 60 years. The risk of death following COVID-19 was substantially higher after ASCT compared to the general population for the same age groups (14). For more comparisons with the general population of Sweden, see Table 3. It should be pointed out that the comparison between the study cohort and the general population is retrospective and merely descriptive. There are several parameters influencing the risk of contracting SARS-CoV-2 that this study cannot cover, including level of self-isolation, use of protective barriers, and level of local community spread.

**Table 3:** Risk of hospitalization, intensive care, and death in ASCT-patients versus the general population.

Age group, years	Cases	Hospitalized, *n* (%)	ICU, *n* (%)	Death, *n* (%)
40–49				
Sweden	**148,656**	5,817 (3.91)	614 (0.41)	90 (0.06)
Study cohort	**5**	1 (20)	0	0
50–59				
Sweden	**133,053**	9,468 (7.13)	1,383 (1.04)	329 (0.25)
Study cohort	**2**	1 (50)	0	0^†^
60–69				
Sweden	**69,354**	10,582 (15.26)	1,890 (2.73)	854 (1.23)
Study cohort^‡^	**10**	4 (40)	2 (20)	3 (27)^§^
Total (40–69)				
Sweden	**351,063**	**25,885** (**7.37**) (CI: 7.29–7.46)	**3,887** (**1.11**) (CI: 1.07–1.14)	**1,273** (**0.36**) (CI: 0.34–0.38)
Study cohort	**17**	**6** (**35**) (CI: 17.3–58.7)	**2** (**12**) (CI: 3.29–34.3)	**3** (**15**) (CI: 5.24–36.0)¶

The table shows the risk of hospitalization, need for ICU, and death following infection with SARS-CoV-2 in the Swedish general population and the study cohort of patients treated with ASCT for hematological malignancy. The percentages shown are compared with the total number of cases for the row. Data from the public health agency of Sweden.

† Mortality data available for four patients in the 50–59 cohort.

‡ One patient was 70 years and 1 month at the time of infection.

§ Mortality data available for 11 patients in the 60–69 years cohort.

¶ Mortality data available for all 20 patients.

Bold numbers indicate total cases of COVID-19 for each row and each column in Sweden and in the study cohort.

In observational studies from the spring of 2020, the COVID-19-related mortality among hematological patients was 31–37%, and among ASCT patients, it was 17–34% with the limitations of primarily including hospitalized patients (11–13). Retrospective register-based studies have later added to the picture. A study of 146 patients treated with ASCT and reported to the EBMT register showed a 6 week overall mortality rate of 27.9% (15). An international observational study of 134 ASCT patients reported to the Center for International Blood and Marrow Transplant Research (CIBMTR) showed an overall mortality rate of 33% at 30 days following COVID-19, with a higher risk for lymphoma patients (16). One reason why our study shows lower mortality rates than previous studies could be that we have managed to include relatively more patients with mild COVID-19. Primarily, this study included ambulatory non-hospitalized patients, which was not the case in most of the other studies. Also the excellent coverage of the Swedish database for SARS-CoV-2 positive tests, due to centralized and mandatory reporting, minimizes the risk of underestimating the incidence of COVID-19.

The risk of hospitalization was 35% and the need for ICU was 12%, which are roughly five and 10 times greater than the risk in the general population, respectively (14). The need for oxygen was reported as 62% in SARS-CoV-2-infected hematological patients (17) in a large previous study, compared to 24% in this study.

Our study suggests that patients with hematological malignancies treated with ASCT have a high risk of a severe course of COVID-19. The immunocompromising effect of the underlying disease and the treatment contribute, most probably, to this prognosis. Though this study was performed during times of uncontrolled spread of the disease, when vaccines were not available and in a country with a liberal approach regarding lockdown and isolation, the absolute risk of COVID-19-related mortality following ASCT was 0.45%.

The incidence of COVID-19 was 4.5% among the ASCT patients in Sweden for the study period. The average time of follow-up for COVID-19 infection was 8.4 months. The majority of the included patients underwent ASCT before the second wave of COVID-19. The incidence of COVID-19 in the general population of Sweden from the first official case on 31 January 2020 to 31 March 2021 was 8.0%. The average monthly incidence in the study population was 0.54% (CI: 0.38–0.82) compared to 0.572% (CI: 0.571–0.573) in the general population, suggesting that the susceptibility to be infected with SARS-CoV-2 is not increased after ASCT.

The strength of this study is the coverage of both ASCT patients and COVID-19 cases in the study cohort as well as nationally, minimizing selection bias. As a result, this study presents relatively more patients with mild symptoms compared to previous studies, making the results reported a reliable description of the impact of COVID-19 on ASCT patients on a national level.

The main limitation of this study is the limited number of SARS-CoV-2 positive patients in the cohort; hence, discussion on associations between risk factors, complications, and outcome is not statistically meaningful. Another limitation was the hesitation to perform ASCT in the spring of 2020. Exactly which and how many patients who were affected are not known. For comparison, we identified 442 unique patients who were treated with ASCT in 2020 in Sweden, compared to 460 unique patients reported to the EBMT in 2019.

Due to the limited availability of PCR tests in Sweden in early 2020, the number of individuals who contracted SARS-CoV-2 during that time may have been underestimated. PCR tests were available for patients who needed hospital care and presented with symptoms of COVID-19. Among patients with mild symptoms, it is likely that patients with malignancies were prioritized for testing. This may contribute to an overestimation of the risk of infection with COVID-19 in the study cohort compared to the general population. On the other hand, the same principle could overestimate the risk of severe disease in the general population. The scale of the missing cases is unknown. In addition, one can argue that ASCT patients would be more vigilant in regard to self-isolation and risk-mitigation, even though they needed repeated visits to the hospital.

As none of the patients in this cohort had been vaccinated for SARS-CoV-2, the study cannot evaluate how vaccination affects the outcome of the disease. Furthermore, there is a continuous evolution of new variants of SARS-CoV-2; thus, the results from this study could be less applicable if new variants of different virology and clinical severity were to dominate the spread of disease.

In conclusion, COVID-19 constitutes a higher risk of mortality and hospitalization for ASCT-treated hematological cancer patients compared to the general population, though the risk of contracting SARS-CoV-2 is, most probably, comparable. The risk of death, and the need for hospital care, oxygen, and intensive care seem lower in this study compared with previous studies (11–13, 15–17). The absolute risk of COVID-19-related mortality after ASCT was less than 0.5%. This study suggests that the COVID-19 pandemic is not a contraindication for ASCT as long as the treatment is clinically motivated, patients are well educated, and prophylactic measures are followed.

## Ethical approval

Approval from the Ethical Review Agency in Sweden was granted with the identification number EPN Dnr 2020-01781 on 23 April 2020, with amendment on 3 August 2020 with identification number EPN Dnr 2020-03433. All patients have given written consent to allow their data to be reported to the EBMT.

## Data sharing

Individual participant data that underlie the results reported in this article, after de-identification (text, tables, and appendix) will be available together with the study protocol from 9 to 24 months following article publication. Proposals should be directed to thomas.silfverberg@medsci.uu.se; to gain access, data requestors will need to provide a draft of a data access agreement that will be evaluated.

## Supplementary Material

Impact of COVID-19 on patients treated with autologous hematopoietic stem cell transplantation: A retrospective cohort study

## References

[1] Phelan AL, Katz R, Gostin LO. The novel coronavirus originating in Wuhan, China: challenges for global health governance. JAMA. 2020;323:709–10. doi: 10.1001/jama.2020.1097. 31999307

[2] He W, Chen L, Chen L, Yuan G, Fang Y, Chen W, et al. COVID-19 in persons with haematological cancers. Leukemia. 2020;34:1637–45. doi: 10.1038/s41375-020-0836-7. 32332856 PMC7180672

[3] Dai M, Liu D, Liu M, Zhou F, Li G, Chen Z, et al. Patients with cancer appear more vulnerable to SARS-CoV-2: a multicenter study during the COVID-19 outbreak. Cancer Discov. 2020;10:783–91. doi: 10.1158/2159-8290.CD-20-0422. 32345594 PMC7309152

[4] Ljungman P, Mikulska M, de la Camara R, Basak GW, Chabannon C, Corbacioglu S, et al. The challenge of COVID-19 and hematopoietic cell transplantation; EBMT recommendations for management of hematopoietic cell transplant recipients, their donors, and patients undergoing CAR T-cell therapy. Bone Marrow Transplant. 2020;55:2071–6. doi: 10.1038/s41409-020-0919-0. 32404975 PMC7220575

[5] Terpos E, Engelhardt M, Cook G, Gay F, Mateos MV, Ntanasis-Stathopoulos I, et al. Management of patients with multiple myeloma in the era of COVID-19 pandemic: a consensus paper from the European Myeloma Network (EMN). Leukemia. 2020;34:2000–11. doi: 10.1038/s41375-020-0876-z. 32444866 PMC7244257

[6] The Public Health Agency of Sweden (Folkhälsomyndigheten). Veckorapport om covid-19, vecka 13 2021. Available from: https://www.folkhalsomyndigheten.se/globalassets/statistik-uppfoljning/smittsamma-sjukdomar/veckorapporter-covid-19/2021/covid-19-veckorapport-2021-vecka-13-final.pdf [cited 11 May 2021].

[7] The Public Health Agency of Sweden (Folkhälsomyndigheten). Veckorapport om COVID-19, vecka 9 2021. Available from: https://www.folkhalsomyndigheten.se/globalassets/statistik-uppfoljning/smittsamma-sjukdomar/veckorapporter-covid-19/2021/covid-19-veckorapport-2021-vecka-9-final.pdf [cited 11 May 2021].

[8] Statistics Sweden (Statistika Centralbyrån). Population in the country, counties and municipalities on December 31, 2020 and population change in October–December 2020 2021. Available from: https://www.scb.se/en/finding-statistics/statistics-by-subject-area/population/population-composition/population-statistics/pong/tables-and-graphs/quarterly-population-statistics--municipalities-counties-and-the-whole-country/quarter-4-2020/ [cited 11 May 2021].

[9] World Health Organization. International guidelines for certification and classification (coding) of COVID-19 as cause of death 2020. Available from: https://cdn.who.int/media/docs/default-source/classification/icd/covid-19/guidelines-cause-of-death-covid-19-20200420-en.pdf?sfvrsn=35fdd864_2 [cited 4 February 2021].

[10] Characterisation WHOWGotC, Management of C-i. A minimal common outcome measure set for COVID-19 clinical research. Lancet Infect Dis. 2020;20:e192–7. doi: 10.1016/S1473-3099(20)30483-7 32539990 PMC7292605

[11] Passamonti F, Cattaneo C, Arcaini L, Bruna R, Cavo M, Merli F, et al. Clinical characteristics and risk factors associated with COVID-19 severity in patients with haematological malignancies in Italy: a retrospective, multicentre, cohort study. Lancet Haematol. 2020;7:e737–45. doi: 10.1016/S2352-3026(20)30251-9 32798473 PMC7426107

[12] Pinana JL, Martino R, Garcia-Garcia I, Parody R, Morales MD, Benzo G, et al. Risk factors and outcome of COVID-19 in patients with hematological malignancies. Exp Hematol Oncol. 2020;9:21. doi: 10.1186/s40164-020-00177-z 32864192 PMC7445734

[13] Vijenthira A, Gong IY, Fox TA, Booth S, Cook G, Fattizzo B, et al. Outcomes of patients with hematologic malignancies and COVID-19: a systematic review and meta-analysis of 3377 patients. Blood. 2020;136:2881–92. doi: 10.1182/blood.2020008824 33113551 PMC7746126

[14] The Public Health Agency of Sweden (Folkhälsomyndigheten). Statistik och analyser om covid-19 inklusive vaccinationer 2021. Available from: https://www.folkhalsomyndigheten.se/smittskydd-beredskap/utbrott/aktuella-utbrott/covid-19/statistik-och-analyser/ [cited 20 December 2021].

[15] Ljungman P, de la Camara R, Mikulska M, Tridello G, Aguado B, Zahrani MA, et al. COVID-19 and stem cell transplantation; results from an EBMT and GETH multicenter prospective survey. Leukemia. 2021;35:2885–94. doi: 10.1038/s41375-021-01302-5 34079042 PMC8171362

[16] Sharma A, Bhatt NS, St Martin A, Abid MB, Bloomquist J, Chemaly RF, et al. Clinical characteristics and outcomes of COVID-19 in haematopoietic stem-cell transplantation recipients: an observational cohort study. Lancet Haematol. 2021;8:e185–93. doi: 10.1016/S2352-3026(20)30429-4 33482113 PMC7816949

[17] Cattaneo C, Daffini R, Pagani C, Salvetti M, Mancini V, Borlenghi E, et al. Clinical characteristics and risk factors for mortality in hematologic patients affected by COVID-19. Cancer. 2020;126:5069–76. doi: 10.1002/cncr.33160 32910456

