# Supplementary material for: Impact of COVID-19 on patients treated with autologous hematopoietic stem cell transplantation: A retrospective cohort study
Source: Ups J Med Sci. 2022 Aug 25;127:10.48101/ujms.v127.8611. doi: 10.48101/ujms.v127.8611 (PMC9447418; doi:10.48101/ujms.v127.8611)
Supplement: Impact of COVID-19 on patients treated with autologous hematopoietic stem cell transplantation: A retrospective cohort study [file UJMS-127-8611-s001.pdf]

SUPPLEMENT 1. SECONDARY ENDPOINTS

**Level of supportive care**
• Need for and duration of hospitalization
• Need for and duration of oxygen treatment
• Need for and duration of high-flow oxygen therapy or non-invasive ventilation
• Need for and duration of intensive care
• Need for and duration of invasive mechanical ventilation or ECMO
**Occurrence of complications**
• Acute respiratory distress syndrome (ARDS)
• Arterial and venous thrombosis
• Verified arrhythmias
• Acute cardiac injury
• Stroke
• Secondary infection
• Cytokine release syndrome (CRS)
**Any significant risk factor for severe outcome**
• Comorbidities†
• Underlying disease including status of disease
• Previous disease modifying treatment in the last six months
• Conditioning regimen
• Age and gender
• Time in relation to ASCT
• Neutropenia
• Laboratory risk factors‡

†Comorbidities included cardiovascular disease, diabetes mellitus type I and II, hypertension, chronic lung disease, chronic kidney disease, stroke, chronic liver disease, previous organ or allogeneic/autologous hematopoietic stem cell transplantation, other active cancer, obesity and smoking). ‡Laboratory risk factors included elevated C-reactive protein, low leukocyte counts, low lymphocyte count, elevated liver enzymes above normal, lactate dehydrogenase, ferritin, d-dimer, activated partial thromboplastin time (aPTT), troponin and creatinine.
